# Visual form of ASL verb signs predicts non-signer judgment of transitivity

**DOI:** 10.1371/journal.pone.0262098

**Published:** 2022-02-25

**Authors:** Chuck Bradley, Evie A. Malaia, Jeffrey Mark Siskind, Ronnie B. Wilbur

**Affiliations:** 1 Department of Linguistics, Purdue University, West Lafayette, Indiana, United States of America; 2 Department of Communicative Disorders, University of Alabama, Tuscaloosa, Alabama, United States of America; 3 Elmore Family School School of Electrical and Computer Engineering, Purdue University, West Lafayette, Indiana, United States of America; 4 Department of Speech, Language, and Hearing Sciences, Purdue University, West Lafayette, Indiana, United States of America; University of Birmingham, UNITED KINGDOM

## Abstract

Longstanding cross-linguistic work on event representations in spoken languages have argued for a robust mapping between an event’s underlying representation and its syntactic encoding, such that–for example–the agent of an event is most frequently mapped to subject position. In the same vein, sign languages have long been claimed to construct signs that visually represent their meaning, i.e., signs that are iconic. Experimental research on linguistic parameters such as plurality and aspect has recently shown some of them to be visually universal in sign, i.e. recognized by non-signers as well as signers, and have identified specific visual cues that achieve this mapping. However, little is known about what makes action representations in sign language iconic, or whether and how the mapping of underlying event representations to syntactic encoding is visually apparent in the form of a verb sign. To this end, we asked what visual cues non-signers may use in evaluating transitivity (i.e., the number of entities involved in an action). To do this, we correlated non-signer judgments about transitivity of verb signs from American Sign Language (ASL) with phonological characteristics of these signs. We found that non-signers did not accurately guess the transitivity of the signs, but that non-signer transitivity judgments can nevertheless be predicted from the signs’ visual characteristics. Further, non-signers cue in on just those features that code event representations across sign languages, despite interpreting them differently. This suggests the existence of visual biases that underlie detection of linguistic categories, such as transitivity, which may uncouple from underlying conceptual representations over time in mature sign languages due to lexicalization processes.

## Introduction

There are strong cross-linguistic tendencies for verbal arguments with particular semantic roles to surface in particular syntactic positions, e.g., agents are often subjects [1, 2], or for verbs with certain meanings to be coded with a particular argument structure [3, 4], e.g., events denoting transfer are often ditransitive, independent of modality (i.e., signed vs. spoken) [5, 6]. However, the association of thematic roles, conceptual structure and argument structure is invisible in spoken languages. In spoken languages the argument structure of a verb or the thematic roles it assigns are opaque with respect to overt phonological form or morphological marking; instead, argument structure can be deduced from its syntactic distribution. For example, the English words *eat*, *dine*, and *devour* all roughly describe the same event insofar as there is an agent who ingests a theme, whether stated or not. However, the three differ with respect to their syntactic frames, with *eat* being ambitransitive (*Omar ate [the salad]*), *dine* being obligatorily intransitive (Omar dined [*the salad]), and *devour* being obligatorily transitive (Omar devoured *[the salad]). There is nothing in the phonological structure of these words that suggest how many arguments they select for. With respect to morphological marking, in some languages, like Turkish, the addition of causative morphology distinguishes transitive from intransitive frames for the same basic event (cf. 1a, 1b). Of course, there also exists valency-reducing morphology, as in the case of anticausative and passive marking [7]. However, it is unknown to what extent valency-changing morphology is transparent, such that even people unfamiliar with a given language would be able to infer the argument structure of a verb on first exposure.

(1)
  a Kalem   düş-tü-Ø
    pencil.NOM fall.PST-3S
    ‘The pencil fell’
  b Çocuk   kalem-i   düş-**ür**-dü
    child.NOM pencil-ACC fall-**CAUS**-PST.3SG
    ‘The child dropped/made fall the pencil’

However, sign languages have been known to manifest aspects of event representations and logical form overtly in the phonological form of signs [8]. For example, pointing signs in sign languages have been analyzed as the overt manifestations of referential loci [9], where points in space are standardly associated with unique individuals (but see [10] for an important refinement). This iconicity theoretically allows conceptual structure and meaning to be accessible to those unfamiliar with sign languages via phonological form: Non-signers have been shown to spontaneously produce and accurately interpret gestures that establish coreference using strategies that are visually similar to reference tracking and agreement marking in sign languages [11–13].

With respect to argument structure, specifically, sign languages exhibit a few characteristic ways in which arguments are overtly marked. In the classifier system of sign languages, a system of highly iconic constructions expressing motion events and events of manipulation, event participants are mapped to specific visual forms. For instance, Benedicto and Brentari [14] show that aspects of handshape encode agents and themes in American Sign Language: Agents are mapped to a handpart morpheme, which specifies the relative orientation of the palm, fingertips and other parts of the hand, and themes are instead mapped to selected fingers. Benedicto and Brentari’s analysis has since been shown to hold for geographically and genealogically unrelated sign languages, demonstrating that the use of handshape to encode verb valency is robust (for American Sign Language (SL): [14]; ASL, Argentine SL, and Catalan SL: Benedicto et al., [15]; Italian SL, ASL, Nicaraguan SL, Nicaraguan homesigners: [16]; SL of the Netherlands SL: [17]; Turkish SL: [18]; *inter alia*.).

Further, each hand of a classifier construction may manifest a unique event participant, with the interaction between the hands specifying the nature of the event [19]. The movement of the dominant hand towards the non-dominant hand, for instance, may encode events of *approaching*, *passing* or *hitting*, where the agent is mapped to the dominant hand and the goal, via or theme roles are mapped to the non-dominant hand, respectively, depending on whether the two hands are in the same plane or achieve contact [20, 21]. Similar patterns have been found within lexical verbs in sign languages: In a cross-linguistic study of 31 sign languages, Östling et al. [22] found that plural concepts are significantly more likely to be expressed by two-handed signs over one-handed signs. These concepts include events that involve more than one participant, such as *argue*, *compare*, *be similar* and *fight*, further suggesting a close connection between an event’s underlying conceptual structure and its phonetic and syntactic realizations in sign languages.

As a final example, overt expressions of conceptual structure have been also documented within the argument-marking systems of sign languages: Concepts denoting literal or metaphorical transfer, like *give* and *inform*, tend to be coded with verb movement within and across sign languages [23–25]: The verb begins at the locus associated with the source thematic role and terminates at the locus associated with the goal thematic role (termed *directionality*; see INFORM at https://asl-lex.org/visualization/?sign=let_know). Events that do not denote transfer, like *film (a movie)* and *break*, may visually represent arguments using a different strategy (FILM in ASL marks objects with palm/fingertip orientation; https://asl-lex.org/visualization/?sign=film) or not at all (BREAK in ASL does not mark arguments; https://asl-lex.org/visualization/?sign=break).

In sum, while there is language-internal and cross-linguistic variation in the precise way that conceptual structure is ultimately mapped to syntactic and phonological form in sign languages, handshape marking, directionality, and the mapping of event participants to each hand are common, robust strategies for argument realization. One explanation of this robustness is the hypothesis that they have domain-general cognitive underpinnings, which are iconically expressed in the form of signed verbs.

Despite the invocation of iconicity to explain why signs resemble their meanings, few studies have objectively measured and quantified iconicity by having participants unfamiliar with a sign language infer linguistic possibilities about that language (e.g., the transparency of the encyclopedic content of signs; [26–28]). Further, fewer still have quantified the specific visual characteristics that undergird this iconicity. In one inaugural experimental study of the mapping between conceptual structure and overt (morpho)phonological form in sign language, Strickland et al. [29] demonstrate that non-signers are sensitive to a visual representation of event boundaries, or telicity, in sign languages, independent of specific semantics of the verb, or the sign language under observation. Naïve observers are also able to segment real life events into subevents using a common set of cognitive heuristics based on motion kinematics in the scene [30], which are similar to the kinematic features that distinguish signs with and without event boundaries in different sign languages (cf. ASL [31, 32]; Croatian Sign Language [33]). Thus, there is mounting evidence for the existence of a universal mapping bias between visual cues and conceptual representations of events across sign languages.

In this study, we examine the lexical feature transitivity, or how many arguments a verb takes. We aim to discover what may be universally available mapping biases between visual form and conceptual representations, noting that language experience may alter the perception of iconicity [27, 34, 35]. In a transitivity judgment study and feature-based modeling analysis, we explore the question of whether transitivity distinctions are manifested in the phonetics (visual form) of ASL lexical signs and, as such, have their basis in perception. If this is the case, transitivity distinctions should be available from visual form to non-signers unfamiliar with the language. In so doing, we hope to demonstrate that cognitive biases shared by both signers and non-signers may help explain how structure arises in a new sign language. We first assess whether non-signers are consistent as a group in identifying ASL verbs’ transitivity status (broadly: transitive and intransitive; and more fine-grained: transitive, ditransitive, intransitive unergative, or intransitive unaccusative). If non-signers are consistent in how they label verbs, there is evidence to support the conclusion that they build a model of transitivity of ASL lexical verbs based on visual form, despite not having access to their lexical properties. We then consider the question of which visual features guide transitivity determinations. If transitivity classing is guided by perceptual features, we would expect the phonetic characteristics of signs to be predictive of non-signer transitivity judgments. We test this hypothesis by correlating visual features from ASL-LEX [36], a lexical database of ASL signs, and some coded by the authors with non-signer transitivity judgments. The analysis revealed that non-signers were generally inaccurate at guessing the transitivity of the signs, but that several visual parameters related to handshape and place of articulation guide non-signers in distinguishing transitivity classes. At the same time, these visual parameters (handshape, place of articulation) are relevant to the encoding of events across sign language lexica; non-signers did not use features irrelevant to event encoding in their determinations. We suggest that the organization of these features in ASL may have shifted away from such visual biases over time due to lexicalization processes.

## Materials and methods

### Participants

A total of 148 participants recruited from Amazon Mechanical Turk (AMT) participated in the transitivity judgment task. We collected minimal demographic information, including participants’ hearing status, family hearing status, familiarity with a sign language, vision information, subjective English fluency, and place of residence (via IP address). We used this information as follows: We used the questions about history with a sign language to exclude participants’ responses. Participants’ vision status and subjective English fluency were used to assess whether participants could properly understand the instructions and view the stimuli. All participants reported normal or corrected-to-normal vision and competency in English. Finally, we restricted the experiment to those participants logging in from the United States, but otherwise did not use place of residence in the analysis. The study has been approved by the Purdue IRB (IRB #1703018974).

### Stimuli

All 197 verbs from the ASL-LEX 1.0 database [36] were used in the study. Videos varied in length between 1 and 4 seconds, and always depicted the same woman in front of a blue backdrop signing one individual sign. The videos can be viewed at asl-lex.org.

In addition, we included three comprehension videos and one foil video. The comprehension videos were included to assess whether participants understood the task. These videos all depicted real life actions. One was intended to be intransitive, and depicted a block tower collapsing. One video was intended to be transitive, and depicted a person hammering a nail into a wooden box. The last was intended to be ditransitive and showed two people exchanging business cards.

Based on a pilot study using these live action videos, we expected that participants should uniformly select appropriate target labels for each comprehension video. The foil item was included to ensure that participants were paying attention. This item was a video that displayed the text “Please choose response (b).” In all, there was thus a low probability that a participant would answer each comprehension question and the foil trial correctly if they were randomly choosing options without viewing each video.

Finally, to be sure that participants could not determine which items contained comprehension or foil videos at first blush, we hid each video behind a poster (a light pink jpeg image), which disappeared as soon as participants hit ‘play.’

### Design and procedure

Participants were asked to decide whether the action denoted by the verb involves:

1. Someone/something is acting on someone/something else
2. An object changes possession or is placed somewhere
3. Something changes shape or location
4. Someone is performing an action without an object

Here, option 1 was coded as a transitive event, 2 as a ditransitive event, 3 as an unaccusative intransitive event, and 4 as an unergative intransitive event. Before the experiment began, participants were given several example verbs that have similar meanings to each option. For example, the verbs *grabbing*, *picking up*, *hitting*, *squeezing* can all be described by option 1. Then, participants were given one example item: The item contained the video BREAK, the classification of BREAK as a transitive verb, and a short justification for why that answer was selected. The text of the justification read, “Here, we chose option 1 since someone (the woman) appears to be acting on (breaking) something (a stick).” This explanation was meant to calibrate the participants towards how we wanted them to think about the experimental items, though participants did not have to provide justifications for their selections. The experiment immediately followed.

We split the survey into six smaller surveys, five with 33 lexical verbs and one with 32, such that no participant saw all 197 verbs. The items included in each survey did not significantly differ from each other with respect to length, frequency, and other lexical features (see S1 and S2 Tables). Three lexical verbs per survey (four in the case of the 6th survey) were repeated in another survey. That is, for example, Survey 2 contained three verbs from Survey 1; Survey 3 three verbs from Survey 2; and so on. This was to ensure consistency in rating across surveys and justify treating all the survey takers as a single population, rather than six disjoint populations. In this way, there were 19 repeated items for a total of (197 + 19 =) 216 lexical verbs included across all surveys. Per survey, there were 40 items (i.e., 32–33 test items, three comprehension items, three to four repeated videos and one foil video). At the end of the survey, participants were asked demographic questions and were then given the opportunity to read a consent statement. We considered the submission of the survey to be participants’ consent to participate in the survey. On average, the surveys took 21 minutes to complete.

To note, although our study is interested in the perception of transitivity in ASL lexical signs, we chose to use semantic descriptions of verb frames rather than specific syntactic definitions for a few reasons. The first was to avoid technical linguistic terms, like *transitive* and *unergative*, which themselves require the understanding of *direct object*, *adjunct*, and *agent* to adequately grasp. Second, it has recently been argued that iconicity principles do not necessarily map to particular syntactic structure, but onto conceptual structure itself. For instance, Kuhn et al. [37] show that non-signers can detect *boundedness* in signed verbs denoting telic events or count nouns via the visual properties of the signs. Signs that involve the deceleration of the hand(s) towards a point, thus indicating a boundary, were more likely to be perceived as denoting count nouns or telic events. That this phonetic cue maps to *boundedness* and not telicity or counthood illustrates that the cue is not bound to a particular syntactic structure (i.e., noun phrase or verb phrase), but rather targets conceptual structure. Further, the cue is not associated with count nouns or telic events that do not involve boundaries in their conceptual meaning: Non-signers were not more likely to associate perceptual boundaries with abstract count nouns like *idea* over abstract mass nouns like *knowledge*, or for abstract telic events like *choose* over atelic events like *dream*.

From here, the conceptual structure of events and grammatical structure have a probabilistic relationship, with certain mappings being prototypical [38]: Proto-agents, defined gradiently in semantic terms (volitionality, causality, and so on), are prototypical subjects; proto-patients, defined in opposing terms, are prototypical direct objects. Further, experiments on event perception (e.g., those reviewed in [39]) suggest that the conceptual and syntactic structure of events are homologous, mirroring theoretical accounts [3, 40].

### Data processing

#### Participant elimination

Responses were recorded from a total of 148 participants. Responses were downloaded from AMT and processed through a sequence of Python routines to (a) remove non-compliant participants, (b) extract judgments for each item and tally them, and (c) establish a measure of consistency. Specifically, we first removed participants who identified themselves as a signer, or who did not answer this question (n = 16). For the purpose of this study, a ‘signer’ is someone with familiarity with a sign language beyond the alphabet and a few signs. Participants who did not respond as expected to more than one of the comprehension videos were considered to have not understood the instructions and their responses were excluded (n = 4). Five participants failed the foil trial, and were excluded from the analysis. Finally, each individual’s response vector was checked for a biased response pattern. One participant passed the foil and comprehension trials but responded with the same answer to more than 80% of the items (> 29 experimental items). This participant’s data was also excluded from the study. After pre-processing, data from 122 participants remained for further analysis. For experimental trials, the minimum number of responses for an item was 18; the maximum number of responses for an item was 22; and the average number of responses for an item was approximately 20.

#### Verb labeling

Each verb was assigned a category (transitive, ditransitive, unergative or unaccusative) by plurality consensus of the participants. For example, if most participants labeled a verb transitive, then that verb was considered to be transitive. By this method, participants at the group level categorized in a strongly binary fashion, categorizing verbs as transitive or unergative at higher rates than ditransitive and unaccusative (Table 1, Column A). As such, we decided to conduct a second analysis and pool responses into a superordinate object-taking (i.e., transitive) class and a superordinate non-object-taking (i.e., intransitive) class. Here, we tallied transitive/ditransitive responses and unergative/unaccusative responses first, with the superordinate category having the most combined responses labeling the verb. By this method, there was a roughly equal number of transitive responses as intransitive.

**Table 1:** Distribution of non-signer transitivity determinations in four and two categories.

Four categories		Two categories	
Transitive	64	Transitive	94
Ditransitive	20		
Unaccusative	22	Intransitive	97
Unergative	72		
Total	179/179		191/191
Column A		Column B	

**(Column A)** Distribution of non-signer transitivity determinations in all four categories, derived by majority consensus. The total of 179 represents removal of 14 verbs with tied responses. **(Column B)** Distribution of non-signer transitivity determinations in two broader categories. The total of 191 represents removal of 3 verbs with tied responses.

#### Eliminated items

Due to a mismatch between videos in our dataset and the ASL-LEX database, three verbs from our study were excluded, bringing the total number of verbs for analysis to 194. In the analysis where verbs were categorized into four categories, there were 14 items where two options received the same number of responses (e.g., participants chose options 1 and 2 with the same frequency). In the analysis where responses were binned into transitive and intransitive categories, there were just three ties. In each case, we excluded ties from further analysis. At this point, due to the number of items we had to exclude from the analysis with four categories, and due in part to the imbalance between the frequency of each of the four categories, we decided to pursue only the analysis with the two superordinate transitivity categories (i.e., transitive and intransitive).

Finally, to check for consistency across survey forms, we compared the labels of the 19 verbs that were shared between surveys. Twelve of the 19 were consistently labeled, while seven were not. For the seven inconsistently labeled verbs we calculated the proportion of transitive responses. If the proportion of transitive votes was greater than 50%, the verb was labeled transitive. If the proportion was lower than 50%, the verb was labeled intransitive. Thus, after preprocessing, 191 of ASL-LEX’s 197 verbs were included in the analysis.

### Consistency, accuracy in labeling

**Fig 1:**
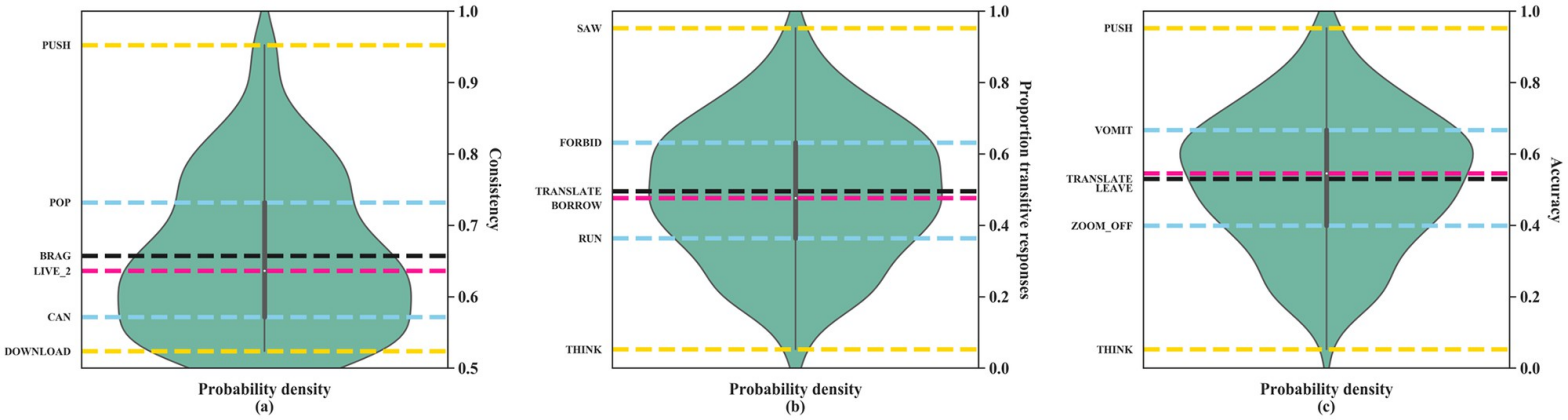
Violin plots showing the distribution of participant responses: Consistency (a), transitivity guesses (b), and accuracy of guesses (c). Width of the violin represents the probability density of the distribution, with wider regions of the plot indicating a higher density of items. In all plots, yellow/light grey dashed lines represent extreme values (max and min), blue/grey lines the upper (75%) and lower quantiles (25%), the pink/dark grey lines the median, and the black/black lines the mean. Example signs are given next to each line, where examples that have values within 1% of each statistic were randomly selected. (a) shows the consistency of non-signer judgments across the dataset. Signs with values closer to 0.5 were more inconsistently judged, while signs with values closer to 1 were more consistently classed; (b) shows the proportion of transitive responses across the entire dataset. Signs with values below 0.5 (BREAKDOWN, LEAVE) were perceived to be intransitive while signs with values above 0.5 were perceived as transitive (TELL, FORBID, PUSH). Signs nearing or at 0.5 were ambiguously transitive or intransitive. By the width of the plot centered around 0.5, most signs were ambiguous, consistent with the plot in (a). (c) shows the proportion of accurate responses, and indicates that non-signers were largely inaccurate at guessing the transitivity of ASL signs.

Overall, participants tended to agree on the transitivity of the verbs they saw. Mean agreement across all verbs was 65.74% (SD: 10.69%). However, non-signers did not agree on the transitivity for a sizable amount of the dataset, indicated by the skewed distribution of consistency shown in Fig 1A. The least consistent signs, including ACCIDENT (‘to crash’), ASK, and EAT, did not seem to form a class, but instead may be characterized by visual cues that clash with respect to transitivity. Some signs that were consistently labeled can be considered ‘motor iconic’, such as SAW (as in ‘to saw wood’), PUSH and PULL (all strongly considered transitive), while others, like THINK, BREAKDOWN, and LAUGH (all strongly considered intransitive) do not have any obvious similarities at first blush.

We did not observe a bias in the proportion of transitive and intransitive judgments. The plot in Fig 1B shows a symmetrical distribution of transitive and intransitive responses. To estimate the non-signers’ judgment accuracy, we then compared the non-signer determinations against the actual transitivity of the signs in ASL (Fig 1C). The transitivity of the signs was determined in consultation with a native signer, who has a Masters degree in linguistics. Following Benedicto & Brentari [14], signs were considered transitive if they could take an object (including obligatorily and optionally transitive verbs, and ditransitive verbs) and could be used with the sign WILLING (i.e., the signs take agentive subjects). Signs not meeting both criteria were considered intransitive. Although we use a categorical definition of transitivity here, future work may benefit from a gradient definition (such as Hopper & Thompson’s [41]), as signs have been demonstrated to express transitivity gradiently [42].

The ASL-LEX database is skewed towards transitive signs, as intransitives only comprise approximately 26% of the database. Thus, given that non-signers did not exhibit a response pattern biased towards transitives (as reflected by the symmetry observed in Fig 1B), they were largely inaccurate at guessing the transitivity of signs: They identified the transitivity of signs correctly at below chance levels (M_acc_ = 0.53, p_chance_ = 0.73), where chance was considered the accuracy achieved if non-signers uniformly chose transitive labels.

Strikingly, the results are at odds with previous studies examining non-signer perception of grammatical phenomena in sign and gesture (e.g., telicity: [29, 37]); distributivity, [43]; phi-features, [13]), which found that non-signers could make accurate inferences. For instance, a study by Strickland and colleagues [29] demonstrated that sign-naïve participants could distinguish telic from atelic events. Further, they found that their participants detected the presence of a gestural boundary (e.g., a sharp deceleration of the hand(s) towards a point in space) in telic signs more so than in atelic signs, which is a robust telicity marker in ASL [44] and other sign languages [33]. As we develop more below, we argue that changes in iconicity over time, the heterogeneity of the concept ‘transitivity,’ and the different ways events are construed may all serve to prevent non-signer transitivity judgments from aligning with the actual transitivity of ASL signs.

We nevertheless argue, that non-signers do have some model of transitivity distinctions based on the features available in the visual signal. From the distribution of transitive and intransitive labels, it appears likely that there are visual features that may guide non-signer judgments. Further, this model of transitivity distinctions is different from a model of actual transitivity encoding, should there be one: These visual cues point non-signers away from the actual transitivity of the signs. We explore where the consistency we observed, irrespective of accuracy, stems from by predicting non-signer judgments from the visual form of the signs used in this experiment.

## Feature-based modeling

Overall, non-signers were consistent in their transitivity judgments, even though they did not guess the transitivity of ASL signs accurately. In this section, we uncover what visual properties of ASL signs guide these judgments.

### Features

Signers take advantage of multiple visual channels in relaying messages, coding linguistic and affective information in different channels simultaneously. For instance, non-manual markers, like eyebrow position, signal that the information conveyed by the hands is a question or statement, among other functions [45]. At the level of individual words, signs can be characterized by combinations of four parameters, handshape, place of articulation, orientation, and movement, with a change in one parameter differentiating between signs (e.g., CANDY and APPLE differ with respect to handshape). In turn, these parameters can be decomposed into parts (e.g., see the handshape decomposition in [46]). Each component or subcomponent is a potential target for iconically-delivered meaning. For instance, signs denoting events of perception (e.g., SEE, SMELL) or body parts (EYES, NOSE) tend to be articulated near relevant body parts across sign languages [47]. That is, there is a connection between place of articulation and lexical meaning of a subset of signs (other signs, like DOUBT, are signed at the eyes but do not have meanings related to perception). Mappings between form and functional meanings are also attested: The movement and contact of the hands, as well as kinematic properties of signs, are recruited for expressing telicity contrasts in ASL [31, 32, 44, 48] and other sign languages [33], which non-signers are able to detect and interpret [29].

To date, though, few studies have looked at the iconic encoding of argument structure in sign languages [5, 14, 16, 42, 49–51] . These studies have found handshape, the number of hands involved in a sign, movement, and place of articulation to be relevant in different subsets of the lexica of ASL and other sign languages. Indirectly, the aforementioned studies on telicity encoding in sign languages may additionally inform the iconic encoding of transitivity insofar as telicity and transitivity conceptually [41] and theoretically [52] overlap. We would expect telicity features, like the contact of the hands with each other or with a plane in the signing space to prime a thematic argument [53].

To our knowledge, no study has yet explored the *perception* of argument structure. As such, in the present study we include features previously identified as marking arguments (e.g., handshape and movement), those that are potentially relevant to argument structure perception (telicity features), and several phonetic features that have no *a priori* connection with transitivity, as it is also possible that non-signers focus on cues that are irrelevant to sign language grammars. The list of included features is presented in Table 2 We describe how we narrow down this set of features in the next section.

**Table 2:** List of candidate features potentially relevant to transitivity perception.

Category	Subcategory	Features	Reference
Sign Type	one-handed	one-handed	
	two-handed	same handshape, different handshape, symmetrical/alternating, other, two-handed	[36, 51, 54]
Location	initial	arm, body, hand, head, neutral	[5, 22]
	final	arm, body, hand, head, neutral	[46]
Movement	movement	back and forth, circular, curved, none, other, straight	
	relative movement	towards initial place of articulation, away from initial place of articulation, n/a; towards body, away from body, n/a	[55, 56]
Handshape	selected fingers	flexion, flexion change, spread change, thumb contact, thumb open, thumb closed	
	unselected fingers	closed, extended, n/a	
	complexity	finger complexity, joint complexity	[46, 57]
Telicity	-	±telic, sign length, ulnar rotation, contact (any), initial contact, final contact, repeated movement	[31, 33, 44, 48]
Lexical	-	iconicity score, number of morphemes	[36]

### Feature selection, model parameters

Each verb in the dataset is potentially characterized by 48 visual features, which were either obtained from ASL-LEX or coded in-house. This count includes individual levels within categorical factors (e.g., the *Head*, *Hand*, *Arm* levels within the *Location* factor each count as an individual feature). However, not all features are representative of the entire dataset (i.e., there are features that apply to only a small subset of verbs). Further, many features are redundant with each other, or are otherwise correlated. Finally, not all features are likely relevant to transitivity perception. As such, we performed feature selection to identify just those features that are (a) common throughout the dataset, (b) independent of other model predictors, and (c) individually most predictive of transitivity judgments.

We first eliminated 14 features that applied to only 20% or fewer (i.e., < 38) verbs in the dataset. We then performed an F-test using *Scikit-Learn*’s *f_classif* function [58], which estimates the degree of linear dependency between each feature and the transitivity labels on an individual basis. Features with higher F-values are likely more predictive of transitivity than those with lower F-values. We also computed a correlation matrix, a pairwise Pearson correlation of each feature, to find pairs of features that are highly correlated with each other. Both a list of features with their F-values and the correlation matrix are reported in S1 and S2 Files. In deciding which features to retain, we used F-values and correlation data, while privileging features already identified as related to transitivity in the literature. Specifically, when removing correlated features, we removed the member of the pair with the lower F-value and lower *a priori* theoretical connection to transitivity. Sixteen features were removed in this way. In two instances, it was difficult to determine which of two correlated features to retain. The Location features, *Head* and *Hand*, and the Sign Type features, *One-handed* and *Two-handed (different handshape)* all had high F-values and are all independently connected to transitivity coding. However, both sets of features were highly correlated with each other. We ultimately chose the location features (*Head* and *Hand*) over the others since they achieved higher F-values. Finally, we sorted the list of features by F-values and chose the top five scoring visual features to include in the model. These were: *Flexion change*, *Closure of the unselected fingers* (*NSF closed*), *Thumb contact* (related to handshape), and *Head* and *Hand* (related to place of articulation). To note, five was selected as the cut-off point, since the 5th element scored nearly twice as much as the 6th.

In addition to selecting features by hand, we also performed feature selection using recursive feature elimination with cross-validation. This was implemented with *Scikit-learn*’s *RFECV* function, using the *LogisticRegression* estimator and the *StratifiedKFold* splitter (n_*splits*_ = 5). The estimator was fit with an intercept, and a weight inversely proportional to class prevalence was added due to a slight class imbalance (i.e., there were more perceived intransitive verbs than transitive verbs in the dataset). No other hyperparameters were changed from default. This process was iterated five times, shuffling the dataset each time, as feature elimination is sensitive to the order in which samples are seen. This process identified *Flexion change*, *Thumb contact*, *Head*, and *Hand* as good candidate features in all iterations. *NSF closed* was identified in only two of the five iterations as a good candidate feature. Thus, we feel confident that the features selected for inclusion in the model are appropriate.

From here, a Logit model was fitted using these five features as regressors, with a binary dependent variable (i.e., ‘1’ transitive, ‘0’ intransitive). No regularization or penalization was used, and an intercept was included. We evaluate the overall fit of our model by comparing it against a model using only the intercept. To assess model coverage, we compare the McFadden’s pseudo-R^2^ statistic of the five-predictor model to a model with minimal feature pruning (namely, only highly correlated features are excluded). We also assess model coverage by fitting the model with only 80% of the data points and predicting the remaining 20% of data points in a round-robin cross-validation paradigm. Again, we compare the prediction accuracy of the five-predictor model against the more inclusive model. All statistical analyses were coded using statsmodels [59] in Python 3.

### Results of modeling transitivity predictions based on visual-phonetic features

**Fig 2:**
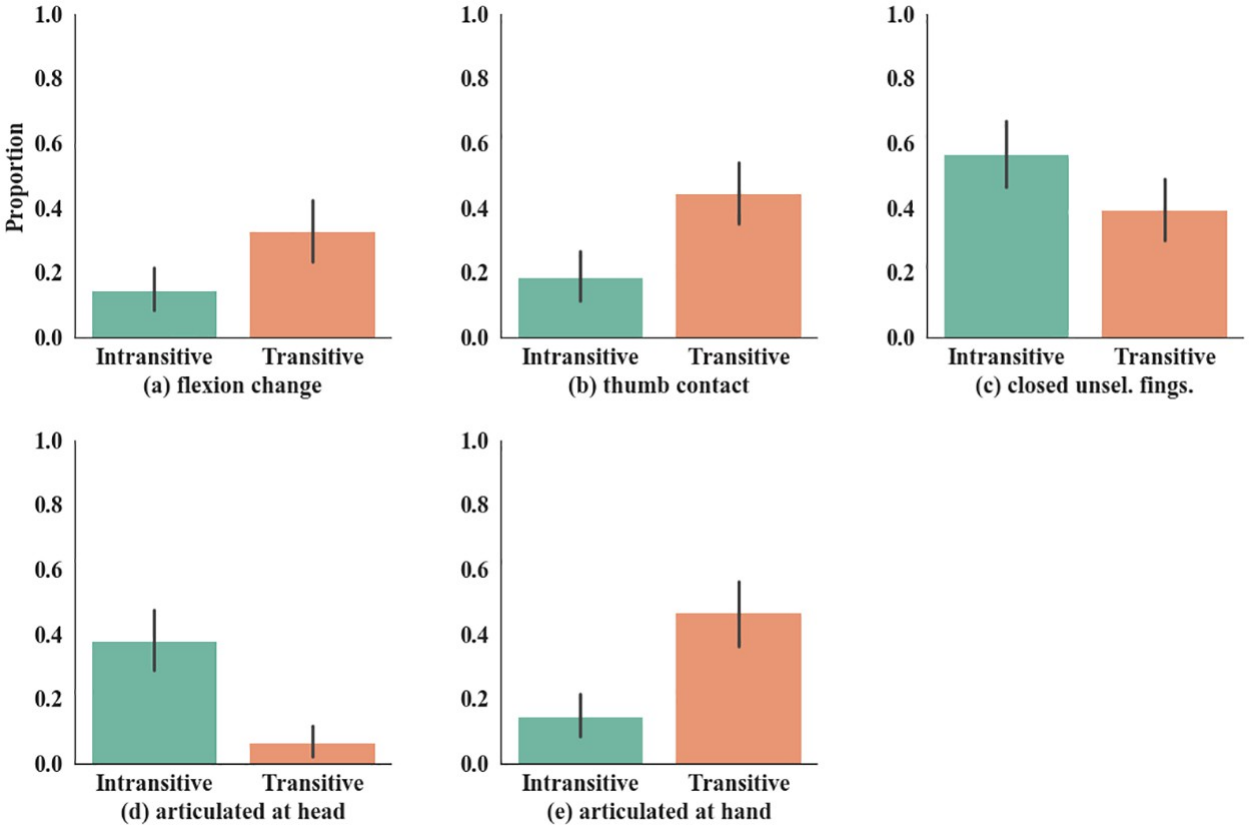
Handshape features. Handshape features (top row): Perceived transitive verbs are characterized by a change in flexion (a) and thumb contact (b). The closure of the unselected fingers was seen to be indicative of intransitive verbs, though not significantly (c). Location features (bottom row): Signs are also more likely to be perceived as transitive if articulated at the non-dominant hand (d). By contrast, verbs articulated at the head were more likely seen as intransitive (e).

A five-predictor model was fitted to predict whether participants viewed ASL lexical verbs as transitive (1) or intransitive (0). Of the five predictors identified through feature selection, two were related to place of articulation of a sign (*Head* and *Hand*), and three were related to the handshape: change of aperture of the hand from closed-to-open or open-to-closed—*Flexion change*, *NSF closed*, and *Thumb contact* (i.e., whether the thumb made contact with at least one finger). All were coded as 1 ‘present’ or 0 ‘absent’. Numerical results are summarized in Table 3, and visualized in Fig 2.

**Table 3:** Table of model predictors.

variable	*β*	SE	Z	p	CI		OR
					0.025	0.975	
intercept	-0.632	0.320	-1.975	0.048	-1.260	-0.005	-0.632
Flexion change	0.970	0.426	2.276	0.023	0.135	1.805	0.970
Thumb contact	1.351	0.408	3.312	0.001	0.551	2.150	1.351
Hand	1.470	0.408	3.600	<0.001	0.670	2.271	1.470
Head	-1.884	0.546	-3.452	0.001	-2.953	-0.814	-1.884
NSF closed	-0.272	0.367	-0.742	0.459	-0.991	0.447	-0.272

The model was significantly predictive of non-signer transitivity judgements (*χ*^2^ = 65.59, df = 5; p < 0.001). Four of the five predictors were significant at p < 0.05, *Flexion change*, *Thumb contact* (handshape), *Hand*, and *Head* (place of articulation), but not *NSF closed* (handshape). A change in flexion, articulation at the hand, and contact of the thumb with the fingers all corresponded with a higher proportion of transitive guesses. On the other hand, signs at the head corresponded with more intransitive guesses.

#### Comparing models with more features

The number of features included in the model is small, such that it is conceivable that much more of the participants’ behavior could be captured by the inclusion of more features. To that end, we compared the performance of the current model (the exclusive model) against a model including 32 features (the inclusive model). Categorical variables were dummy coded. Features that were numerical (i.e., *Iconicity score* and *Sign length*) were scaled by subtracting the mean and dividing by the standard deviation. Similar to before, we removed features that would have resulted in strong collinearity. However, low-frequency features and those with middling F-scores were retained.

The pseudo-R^2^ of the exclusive model is 0.2478, while the pseudo-R^2^ of the inclusive model is 0.3743 (a difference of 0.1265). The increase in the pseudo-R^2^ value indicates that other, less frequent features may be additional avenues of exploration in future studies. While four of the features, *Thumb contact*, *Flexion change*, *Head*, and *Hand* in the exclusive model were all significant predictors in the inclusive model, with the sign of their coefficients pointing in the same direction, *NSF closed* was not significant. No other predictors were significant, though *Iconicity score*, *Ulnar rotation*, and *Initial contact* had p-values between 0.05 and 0.1. (*Iconicity score tended* to typify perceived transitive verbs, while the other two features characterized perceived intransitive verbs).

#### Model coverage using cross-validation

We assessed coverage of the inclusive and exclusive models and then compared them by way of prediction accuracy. We split the dataset (191 verbs) into seven partitions (27 or 28 verbs per partition). We then fit a Logit model (here called a classifier) on just six of the seven partitions (the training set), and had it predict the labels from the seventh, ‘unseen’ partition (the test set). The proportion of intransitive to transitive items in the training and testing sets were as close to the overall proportion of items as in the entire dataset (50.1% of items were intransitive). We evaluated the classifier’s performance using 7-fold cross validation, wherein the splitting, training and testing are performed seven times, each time using a different partition as the test set. For each fold, a new classifier was fit, such that no classifier had information from previous folds. This process was performed twice, once with just five features (exclusive model) and once with 32 features (inclusive model). The results are summarized in Table 4. To note, for the exclusive model, because feature selection was performed on the entire dataset (i.e., prior to the splitting of the dataset into training and testing sets), each classifier in the cross-validation scheme has already ‘seen’ information from the test set. However, at this stage we are not trying to estimate how much transitivity-related information is available in the total signal, but to assess the perceived transitivity information available from the five predictors.

**Table 4:** Accuracy of the exclusive and inclusive models when predicting unseen data.

Fold	1	2	3	4	5	6	7	Avg.
Exclusive	0.75**	0.82***	0.81**	0.67	0.70*	0.56	0.74*	0.72***
Inclusive	0.68*	0.82***	0.70*	0.74*	0.59	0.56	0.63	0.67***

Asterisks indicate that model achieved above chance accuracy, where chance = 0.51.

* p < 0.05,

** p < 0.01,

*** p < 0.001

To calculate significance, we used the cumulative mass function of the binomial distribution. Chance was not 50%, due to a slightly higher proportion of perceived intransitive verbs in the dataset. Thus, we used a blind baseline of p = 0.51, where we assume that the model always chooses the answer that happens to be the most frequent correct answer among all of the trials. For the exclusive model, mean prediction accuracy was 72.29% (std: 0.07; p < 0.001), with five of the seven folds predicting perceived transitivity significantly above chance (see Table 4). On average, the inclusive model was worse at predicting non-signer transitivity determinations, with 66.55% accuracy (std: 0.07, p < 0.001). However, in two folds, the inclusive model scored higher than or tied with the exclusive model. Both models performed poorly (and identically) in the sixth fold. These two observations indicate that (1) participants focused on a specific set of five predictors as relevant to transitivity classing; and (2) there are pockets of the dataset where additional visual features, or features not included in either model, may be more important. From here, we only discuss results derived from the exclusive model.

### Effect of consistency on model coverage

We explored whether non-signers cued in on a subset of signs that all displayed features relevant to transitivity distinctions, by selecting those signs that were judged (a) at or above the median consistency of all items (≈63% consistent), a ‘consistent model’; or (b) below median consistency, an ‘inconsistent’ model. To note, consistency and perceived transitivity did not correlate in any subset of the data (whole dataset: Pearson’s r = -0.09, n.s.; ‘consistent’ model: r = 0.05, n.s.; ‘inconsistent’ model: r = 0.09, n.s.). Ninety-six signs (out of 191) had above median consistency and were included in the ‘consistent’ model. The same five predictors as in the previous analysis were used. The model was significantly more predictive than the intercept-only model (*χ*^2^ = 61.16, p < 0.001), and three of the predictors, *Thumb contact*, *Head*, and *Hand*, were still significantly predictive (and all in the same direction as before). *Flexion change* and *NSF closed* were not significant. Model coverage increased by both measures (pseudo-R^2^ = 0.4682, cf. 0.2478, the pseudo-R^2^ of the model fit with data from all verbs; Mean predictive accuracy = 0.8335, std. = 0.0919, p < 0.001, cf. 0.7229).

For the ‘inconsistent’ model, 95 signs that had below median consistency were included. Again, *Hand*, *Head*, *Thumb contact*, *Flexion change*, and *NSF closed* were included as predictors. The model was again significant (*χ*^2^ = 22.50, p < 0.001), but only one of the predictors, *Flexion change*, was significant. (*NSF closed* approached significance). Model coverage decreased when compared to both the ‘consistent’ model and the model including all items (pseudo-R^2^ = 0.1709; Mean predictive accuracy = 0.6923, std = 0.1303, p < 0.001). Taken together, the performance on the ‘consistent’ and ‘inconsistent’ models suggests that, when present, non-signers used a select few features as a reliable transitivity cue. As we will discuss in more detail below, transitivity is not strongly iconic in the same way throughout the entire ASL-LEX verb dataset.

## Discussion

### Transitivity judgments: Consistency and accuracy

At the group level, participants were consistent in judging the transitivity of ASL signs. Some signs, however, demonstrated a high level of consistency. Of these signs, a roughly equal number were perceived as transitive or as intransitive. The analysis revealed that five visual parameters, three related to handshape and two related to place of articulation, guide non-signers in distinguishing transitivity classes in ASL lexical verbs, especially among the more consistently judged signs.

These findings contribute to a growing body of literature that suggests that iconicity facilitates semantic distinctions not only in sign languages [29], but also in spoken languages (e.g., judgments of size and power, [60]; judgments of shape, [61]; connections between iconicity and word learning, [62, 63]). However, these studies demonstrated that naive participants were accurate in their judgments. In the present study, participants were largely inaccurate in judging the transitivity of ASL signs. That is, the non-signer model of manual transitivity is different from the model actually employed by ASL–if there is one. Non-signer inaccuracy may be attributable to the composition of the ASL lexicon itself.

### Iconic parameters of transitivity

Our exploratory analysis began with a list of 48 features that contrast in the world’s sign languages, from which our feature selection process winnowed the final candidate feature list down to just five. These five features, it turns out, are not random, but independently linked to transitivity encoding in sign languages. This shows that non-signers attend to the same perceptual phenomena that mark transitivity contrasts in sign languages broadly, even if they ultimately do not align with the grammar of ASL, specifically. That is, for instance, non-signers do not utilize curved or circular movement–which have not been tied to transitivity encoding–in their determinations, but instead use the transitivity linked features, Handshape features and Location.

#### Handshape

As two of the handshape-related features, *Flexion change* and *Thumb contact*, are associated with transitive guesses, we propose an embodied explanation: A change in flexion is congruent with the grasping or releasing of an imagined object. For example, the signs THROW and DROP (https://asl-lex.org/visualization/?sign=drop) both involve a change in flexion, as the internal argument is released from a grasp. In other signs, the fingers may be interpreted as enclosing around, crushing or otherwise manipulating an object: The sign WINK, for example, is articulated with the first finger coming in contact with the thumb in front of the eye, as if pinching something. Similarly, the sign GUESS is articulated as if ‘catching’ something in front of the face.

**Fig 3:**
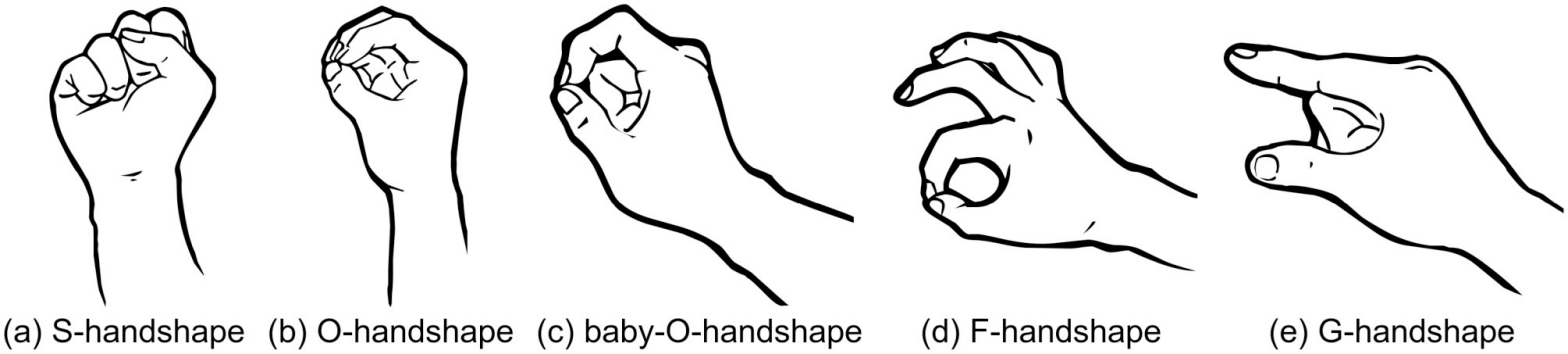
Thumb contact, flexion of unselected fingers. Example ASL handshapes displaying contact between the thumb and the fingers (a–d, but not e). Signs with thumb contact were significantly more likely to be interpreted as transitive than signs without. Handshapes (c) and (d) differ minimally in the flexion of the unselected fingers (in this case, the middle, ring, and pinky fingers). The unselected fingers are closed in (c) but extended in (d). The furling of the unselected fingers tended to characterize intransitive productions across the entire dataset. (To note, since all of the fingers are selected in (a) and (b), there are no unselected fingers.) Finally, each handshape, a–d, is congruent with a type of grasp: (a) and (b) are power grasps, congruent with the agentive movement of or action affecting an object (JUGGLE, IMPACT [‘to hit’]) or tool (SAW [‘to saw’], SHAVE). (c) and (d) are precision grasps, most consistent with tool usage (e.g., WRITE, STIR). Images were generated from the sign language handshape font created by CSLDS at CUHK.

*Thumb contact* may also be an embodied cue. It entails that the thumb and fingers form an enclosure or a fist (Fig 3A–3D vs. 3E), consistent with holding an object. We note that *Thumb contact* is a special case of *Flexion*, or the degree of closure of the fingers (1—‘fully open’ to 7—‘fully closed’), in that signs that display maximum flexion (i.e., ‘closed’/flexion 7) often entail the thumb making contact with the fingers. *Flexion* was also identified as significantly predictive of transitivity class in the F-test pre-processing routine, though it was ultimately dropped due to its high correlation with *Thumb contact*. *Flexion* makes the additional prediction that fully closed handshapes (e.g., Fig 3A are more likely to be considered transitive than mostly closed handshapes (e.g., Fig 3B–3D) and so on (e.g., Fig 3E). This is indeed borne out: 68% of signs with fully closed handshapes (flexion 7) were considered transitive, while fewer signs with a lesser degree of flexion were considered transitive (flexion 6: 64%; flexion 5: 54%; …; flexion 1: 40%). Given the smaller number of examples of signs exhibiting each degree of *Flexion*, it may be the case that Flexion is underpowered. However, the mean flexion rating for signs exhibiting *Thumb contact* is 5.32, while mean flexion of those signs that do not involve thumb contact is 1.98. Future studies, thus, may find that *Flexion* is a more sensitive cue than *Thumb contact*.

The final handshape feature, *NSF closed*, did not reach significance, but tended to characterize intransitive guesses. This is in contrast to Hassemer & Winter’s [64] perception study, where they found that the flexion of unselected fingers is important to non-signers when choosing between a shape or size interpretation in a given handshape. In ‘shape’ interpretations, the authors claim that the selected fingers directly represent an object, while ‘size’ interpretations represent how one would hold an object to demonstrate its size, consistent with intransitive and transitive readings, respectively. The authors found that extended unselected fingers were more likely to signal intransitive interpretations, while closed unselected fingers were more likely to signal transitive readings. We instead found the opposite tendency (closed unselected fingers were more likely to be interpreted as intransitive). This may be due to the fact that selected and unselected fingers were independently manipulated in Hassemer & Winter’s study, while the inventory of ASL handshapes includes (accidental) gaps. For example, while the unselected fingers may be closed or extended in the handshapes in Fig 3C and 3D, the alternate of Fig 3E with extended unselected fingers does not exist in the ASL-LEX database. A greater proportion of signs with extended unselected fingers involved *Flexion*, a strong predictor of transitive interpretations (*Flexion* was not included in the model). The interpretation of closed unselected fingers as denoting intransitive events may be predicated on these facts.

Finally, to support our embodied analysis of handshape perception, we examined a few, specific handshapes. The Baby-O and F- handshapes (Fig 3C and 3D) are both consistent with precision grasps, or the type of handshape required for skillful use of a tool (e.g., SEW, WRITE). The S- and O-handshapes (Fig 3A and 3B) are instead consistent with power grips, the type of handshape required for controlled movement of an object (e.g., EAT, GET). Precision grips tend to be considered transitive more than power grips, where 85.71% of signs with the Baby-O handshape and 75% of signs with the F-handshape were considered transitive versus 73.68% of signs with the S-handshape and 44.44% of signs with the O-handshape were considered transitive (the difference is not significant: t(48) = -1.3960; 1-tailed p = 0.0893). Because precision grips often appear in situations with a prototypical object (*sewing* and *popping* involve the use of a needle-like object) while the association between a power grip and a particular object is not as strong (i.e., the same grip can be used for a wide array of objects and objects may be grasped for transport by many different handshapes), we suggest that this grip-object association may help explain the difference in perception of transitivity.

#### Place of articulation

As to the features related to place of articulation, signs articulated at the head were largely perceived as intransitive, while the contrary is true of verbs articulated at the non-dominant hand. Signs articulated at the hand are all necessarily two-handed signs. Lepic et al. [51] argue that many two handed signs denote some sort of plurality. With respect to transitivity, each hand in a two-handed sign may denote an event participant, with the movement of the sign indicating how the participants interact. For instance, in the sign IMPACT (‘to hit’; https://asl-lex.org/visualization/?sign=impact), the dominant hand represents the agent of the event, or *hitter*, and the non-dominant hand the theme, or *hittee*. As such, we posit that non-signers are sensitive to this iconic strategy. For instance, the sign DROWN is two handed, intransitive, and is articulated such that the second hand resembles a ground (semantically; figuratively, the surface of a body of water). Here, too, each hand represents a different event participant, although only one participant counts with respect to transitivity. Similar signs are CRAWL and ARRIVE. That all of these signs were classed as transitives by non-signers, then, may point to non-signer overapplication of this iconic cue. By contrast, an interactional interpretation is not possible with one-handed signs, which are mostly articulated at the head. This may also help explain why signs articulated at the head were mostly perceived as intransitive.

For *Head* signs, we offer two possible explanations: First, signs articulated at the head are highly correlated with one-handed signs. This could be an intrinsic phonological/organizational principle that signs should be one-handed. It is also observed that many two-handed signs articulated at the head become one-handed over time due to articulatory ease [65]. It could be the case, then, that one-handed signs, lacking an interactional iconicity of two-handed signs (i.e., signs articulated at the hand), may receive a ‘default’ intransitive interpretation. However, one-handed signs are not always articulated at the head and vice versa (correlation between one-handed signs and signs articulated at the head is r = 0.51), so this explanation is incomplete.

Second, verbs signed at the head often denote intransitive events, events that are otherwise low on the transitivity hierarchy [41], or events that have low transitivity prominence (i.e., more frequently occurring as intransitives than transitives; [5, 6, 66, 67]). For instance, verbs of *seeing* and *thinking* are iconically or metaphorically mapped to the eyes and temple in ASL, respectively. Although verbs like SEE (https://asl-lex.org/visualization/?sign=see) and THINK can be transitive, neither event entails that the object is affected and, further, the object of THINK is a proposition. Both facts place these verbs low on the transitivity hierarchy [41]. Non-signers, if sensitive to this more conceptually-linked iconicity, may have thus classed both SEE and THINK as intransitive for this reason.

### Top-down iconicity effects

In the above, we have assumed that perceptual features singly or additively inform non-signer transitivity judgments. That is, representations of grammatical meaning are built bottom-up. However, another possibility is that these features are relevant to guessing the lexical meaning of signs. Then, transitivity is inferred from the lexico-conceptual representation of the meaning. For example, the sign IMPACT (https://asl-lex.org/visualization/?sign=impact) might first be understood to mean ‘hit,’ which often entails both a *hitter* and a *hittee*. In this way, transitivity parses are arrived at from the top down.

This possibility predicts, then, that transitivity parses follow directly from how well participants as a group converge on a meaning for a given sign. As such, consistency in transitivity parses should track consistency in meaning parses: A sign highly depictive of its meaning (e.g., BREAK; https://asl-lex.org/visualization/?sign=break) should have near uniform transitivity parses, while an arbitrary sign (e.g., FILM; https://asl-lex.org/visualization/?sign=film) should have a more inconsistent transitivity parse.

We do not have direct evidence to bear on this point, as we have no way to determine how or whether participants devised lexical meaning from the signs. Nevertheless, we have reason to believe that the top-down approach is less likely than the bottom-up one: First, we conducted an ancillary analysis predicting the consistency of transitivity parses from Iconicity Scores from ASL-LEX. These scores correspond to the degree to which non-signers agree that a sign looks like its English gloss. We found that Iconicity Scores do significantly correlate with the consistency of transitivity parses, but that this correlation does not explain much of the data (F = 14.48, p = 0.0002; R^2^ = 0.071; *β*_*iconscore*_ = 0.0174, p < 0.001). Of course, this analysis also cannot directly assess what lexical meanings non-signers consider when viewing signs. Second, Sehyr & Emmorey [27] obtained Translucency Scores (i.e., non-signer accuracy of guessing a sign in the absence of its meaning) using the same ASL-LEX dataset. Unlike Iconicity Scores, these scores directly assess the meanings non-signers consider. While the authors do not present their item-by-item translucency scores, they report that the scores are quite low and, further, that their participants’ guesses were not very consistent with each other. Altogether, these facts indicate to us that the extraction of transitivity information from signs comes independently of perceived meaning as non-signers are generally poor at converging on a consistent meaning. Naturally, further work is warranted here.

## Implications and conclusion

### Iconicity and the ASL lexicon

Signs are not just iconic or arbitrary, but they are iconic in degrees according to different encyclopaedic or grammatical considerations (and sometimes both). As lexicalization occurs, signers must balance between these different competing sources of iconicity to aid communication. As a language develops, the competition between these factors may shift, or iconic sources may drift to ease production. In the present study, we provide an estimate of the initial visual parameters for transitivity perception in a developing sign community. A follow-up artificial sign language study, manipulating the transitivity features uncovered here, would further constrain and inform this initial estimate.

We note also that patterns at this stage (i.e., first-exposure) may only be weakly present, with systematic interpretation occurring over time. Such has been documented in laboratory experiments on silent gesture transmission, where the form of an initial semistructured silent gesture is constrained by transmission of the signal across different communicators, and the interaction of those communicators [68]. In a developed sign language like ASL, communicative pressures for high information transmission rates and simultaneous error reduction can lead to within-sign constraints on non-dominant handshape and motion, further reducing visible markers of transitivity [69]. Thus, a follow-up study might include signing populations to investigate the reorganization of visual features along the cline of sign language development (i.e., homesigners, and signers of younger and older sign languages).

#### Changes in iconicity

When the hands carry the entire communicative load, certain communicative strategies emerge. The use and form of these strategies change over time, as evidenced from pantomime vs. established sign languages [16, 50, 70], homesign vs. young sign languages vs. established sign languages [71], and different signs or constructions within sign languages (e.g., loan signs and classifier constructions vs. lexical verbs; [72, 73]). These changes may involve the addition, shift, or loss of iconic motivations, along any dimension of iconicity (e.g., lexical or grammatical).

With respect to transitivity, different (sets of) features contribute to signer- and non-signer transitivity labels, or the same features can be used to the opposite effect. For instance, Brentari et al. [16, 50] show that in production, non-signing silent gesturers make transitivity distinctions, though the strategies that they use differ from the strategies employed by the signers in their study. In their 2017 study, the authors found that joint complexity (related to *Flexion*) is higher in transitive than in intransitive pantomimes, and that this is true across American, Chinese, Italian, and Nicaraguan gesturers. Joint complexity in signing groups (including homesigners and signers from young and established sign languages) was lower in transitive than intransitive classifier constructions. That is, signers and non-signers use the same tools (e.g., joint complexity) to the same effect (e.g., in coding transitivity), but use those tools differently in production (a change from a paralinguistic communication system to a linguistic one). Our study adds to this by contributing corroborating data from perception.

Further, signs within sign languages change over time, and may gain or lose iconic elements. For example, Padden et al. [74] and Senghas et al. [75] track the development of directionality–an iconic mechanism that uses space to disambiguate subjects and (indirect) objects–in three young sign languages. At first, signs do not use this iconic strategy. Then, signs begin to inflect for referents that are physically present (gain), before finally inflecting for non-present, and even non-corporeal referents (shift).

**Fig 4:**
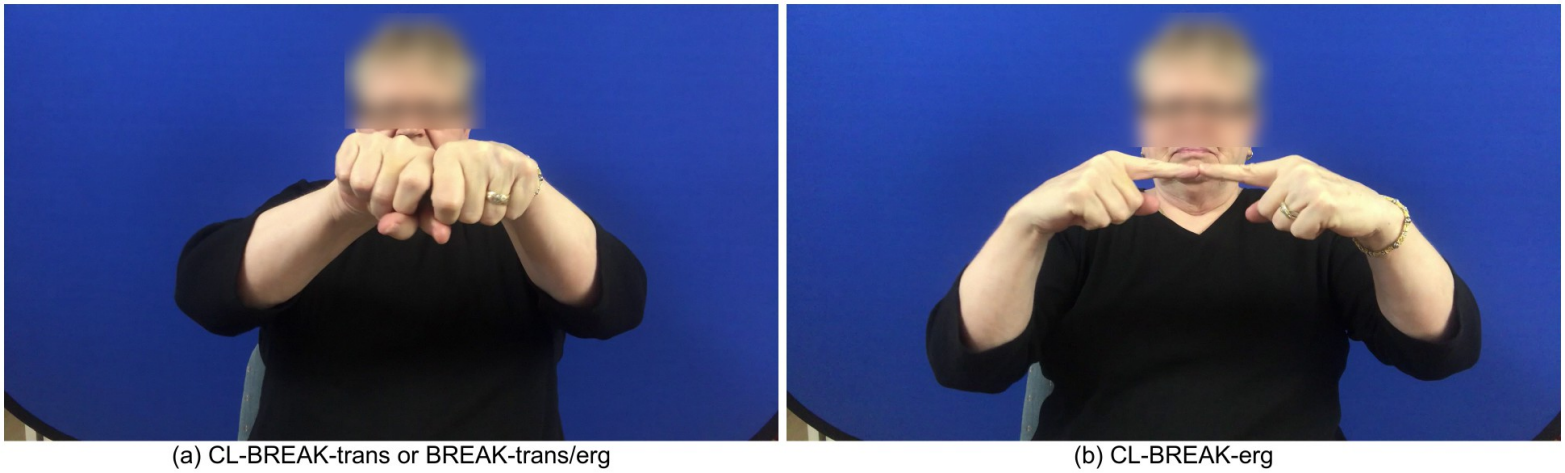
Transitive-unergative distinction in classifier constructions. Three signs meaning break in ASL. (a) and (b) are classifier constructions, within ASL’s classifier system, and are used in transitive (I broke the stick) and unergative (The stick broke) contexts, respectively. A lexical sign, BREAK is historically related to (a) and shares most relevant visual properties. As a lexical sign, BREAK may be used in both transitive and unergative contexts.

As another example, some lexical verbs are derived from classifier constructions. These iconic, multimorphemic constructions are rich in object shape and location information, and may represent transitivity contrasts iconically: For instance, in transitive handling classifier constructions, the hand iconically represents an agent’s hand moving or manipulating a thematic argument. In intransitive entity classifier constructions, the hand represents the single agentive argument. However, classifier constructions may ‘flatten’ to conform to the morphosyntactic [19, 72] and phonological constraints [20, 46, 54] that apply to lexical verbs. For example, the lexical sign BREAK is derived from the transitive classifier construction CL-BREAK-trans[itive] (Fig 4A), where ‘CL’ stands for ‘classifier.’ A different classifier construction for the unergative sense of break exists, and is morphosyntactically and phonologically distinct from the transitive variant [14] (Fig 4B). However, no such lexical verb, say BREAK-erg[ative], exists: Instead, BREAK covers both transitive and unergative events (loss of iconicity). These changes in iconicity (gain, loss, shift) may obscure or misalign transitivity marking strategies–participants would not be expected to be sensitive BREAK being ambitransitive (approximately 85% of respondents classified BREAK as transitive). Another example is the event *eat*, which is also expressed as both a classifier construction and a lexical verb. As a classifier construction, handshape is manipulated to reflect different shape and size attributes of the object: the handshape used for eating apples cannot be used for eating bananas. However, in its lexical form handshape cannot be manipulated at all; the same form is used for both apples and bananas. This loss of motor iconicity perhaps explains why participants judged EAT to be intransitive (although there was considerably less agreement here than with BREAK).

Finally, as suggested earlier, constraints on two-handed signs and syllable structure in ASL may mitigate against visibility of transitivity by obscuring relationships that may be more systematic before lexicalization occurs. Napoli and Wu [56] generalize a number of existing constraints on two-handed lexicalized signs into a single Movement Symmetry Constraint: when both hands move, the position of the hands on their respective paths is either identical or inverse. What might start as a clear movement indicating transitive affect on an object might be lost as the two hands are brought into movement symmetry with each other. Furthermore, when lexicalized, if both hands move, they would have to have the same handshape, otherwise only one of the hands moves and the other serves as the non-dominant hand. If in fact both hands move and have different handshapes, the result is not a verb sign at all but two separate signs, most likely classifier constructions, each carrying separate predicate information and each likely an intransitive verb. Such conditions and constraints interfere with or override transitive iconicity.

#### Heterogeneity of the ASL lexicon

In addition to the historic and synchronic processes that add, shift, or remove iconic strategies, we also take the unexplained variance of the analysis to be reflective of the heterogeneity of the ASL lexicon with respect to iconicity [76]. This is supported by our analysis including only verbs that were consistently classed in that models fitted on a particular subset of the data were more successful than those fitted on the entire dataset. This heterogeneity may stem from, for example, competing iconicities [77], where one type of iconicity (say, lexical iconicity) is selected over another type (e.g., transitivity). In our dataset, WINK (https://asl-lex.org/visualization/?sign=wink), an intransitive verb in both ASL and English, has a mean iconicity rating of 5.75 (non-signers thought it looks like what it means), but was classed as transitive with 73.68% agreement. We believe this is so since the sign is articulated as if pinching a small object in front of the eyes (i.e., a change in aperture). In this case, then, motor iconicity won out over lexical iconicity. As another example, even though GUESS (https://asl-lex.org/visualization/?sign=guess_1) is one handed and signed at the head, two strong predictors of intransitives, there was over 70% agreement that it is transitive. However, the sign is articulated as if one is catching something in front of one’s face. As with WINK, the motor iconicity of the sign (i.e., grasping) outweighs other, generally reliable predictors.

In that same vein, while predicates may be telic or atelic (or be states, processes, achievements, and so on), these represent only a few semantic categories for ASL and other sign languages to represent. And, indeed, sign languages choose visual features that cover all of these distinctions [78]. However, transitivity and its superordinate category, argument structure, are much more nuanced at a lexical level, and separate verbs into many different classes [79]. What makes MEET (interactional/reciprocal-type; https://asl-lex.org/visualization/?sign=meet) transitive is not what makes KILL (kill-type; https://asl-lex.org/visualization/?sign=kill) or KNIT(creation-type; https://asl-lex.org/visualization/?sign=knitting) transitive, which may be represented by (combinations) of different cues. Evidently, the visual characteristics of KNIT are the only ones that cue a transitive interpretation (MEET and KILL were both judged to be intransitive). These classes, if all represented iconically in ASL, would only become apparent in a much larger database.

#### Future directions: Conspiracy of features, competing iconicities, missing features

The model above looked at whether and how individual features from two broad classes of features, handshape and place of articulation, contribute to perceived transitivity. Given the ways in which handshape is operationalized, aspects of handshape features can and do overlap, sometimes additively, with respect to transitivity perception. For example, *Flexion change* and *Thumb contact* were good predictors of perceived transitive verbs individually, as both correlate with transitive guesses (r = 0.22 and r = 0.28, respectively). When combined (i.e., *Flexion change + Thumb contact*), they correlate with transitive guesses in a yet more predictive manner (r = 0.32).

*Flexion change* and *Thumb contact* specifically illustrate the additive value of partially overlapping features (i.e., handshape). However, some transitivity marking strategies may utilize features from two different categories. For example, asymmetrical signs with different handshapes on the dominant and non-dominant hands (as opposed to signs with other Sign Types) correlate with transitive guesses (r = 0.28), as do signs with increasing degrees of flexion (r = 0.24). But, asymmetrical signs with higher degrees of flexion are more likely to elicit transitive guesses than asymmetrical signs with lower degrees of flexion (r = 0.33). Because the feature selection process only identifies features that are individually relevant to transitivity perception, further investigation into the contributions of multiple visual features is warranted.

On the other hand, some articulation parameters in the ASL-LEX database are operationalized in a way that produces conflated features, so analysis of the component subfeatures would be warranted instead. For instance, one feature, Symmetrical or Alternating (related to the interaction and movement of two-handed signs), is true of signs whose movements are mirror symmetrical or are alternating, either of which may be perceived as either transitive- or intransitive-denoting. Napoli and Wu [56] show that different types of symmetries (e.g., rotational, translational) may be active in the morphology of ASL. Further, as discussed above some features are either variations of each other by definition (*Flexion* and *Thumb contact* describe roughly the same concept) or overlap due to the phonological organization of ASL (e.g., few two-handed signs are articulated at the head). This makes uncovering what specific visual characteristic of a sign has the most weight more difficult.

Finally, some features are lacking from the source videos themselves. For instance, directionality marks subjects and (indirect) objects in verbs that either denote transfer, like GIVE and COPY, or metaphorical transfer, like HATE [23, 42, 49]. The videos used in this study (from ASL-LEX [36]) included ‘citation’ or ‘dictionary’ forms of ASL lexical verbs, which do not include directionality. However, evidence from gesture studies indicate that non-signers produce and interpret spatial reference as a subject and (indirect) object marking strategy [8, 11]. Further, Schlenker [80] and Schlenker & Lamberton [81] demonstrate that non-signers associate points in space with distinct discourse referents, which may be referred to anaphorically, but only if these points are made perceptually salient (e.g., through the use of punctuated movement). Although we know of no study that examines non-signer perception of directionality in sign language lexical verbs, these previous studies strongly suggest that directionality would be a meaningful cue. Future work should use videos richer in (potentially relevant) visual features.

## Concluding remarks

While previous work indicated the relationship between visual parameters of hand motion, and conceptual representation of event boundaries [29, 30, 80], the present work extends the study of conceptual interpretation of visual features to event participation; or, in terms of sign language structure, the grammatical feature of transitivity. In this study, we investigated whether transitivity information is perceived in the visual form of ASL predicates. To do this, we gathered non-signer transitivity judgments on the predicates available from the ASL-LEX 1.0 database, and correlated them with the visual characteristics of those predicates. Four visual features, related to handshape and place of articulation, could reliably predict non-signer judgments. The importance of handshape for transitivity perception may be due to an embodied interpretation: Handshapes consistent with holding, releasing, or manipulating an (invisible) object were more likely to be perceived as transitive. Further, the interactional nature of two handed signs (=signs articulated at the hand) may cause non-signers to consider each hand as a distinct event participant. To note, these same visual features do in fact operate iconically within ASL (location: [5]; handshape: [16, 50]; sign type: [51]), suggesting that the form of a sign may be in part determined by iconic considerations with respect to grammatical information.

However, we observed that non-signers were not equally consistent in their judgments across the dataset, and that non-signer judgments often differed from the actual transitivity of the signs. The former may be due to the heterogeneity of the ASL lexicon with respect to iconic strategies, which are sometimes in conflict [76, 77], or the organization of signs into ‘lexical families,’ a family of signs that share some conceptual meaning and most, but not all visual parameters [65, 82]. With respect to accuracy, we suggest that non-signers over-apply iconic strategies to members of the lexicon that do not use them. We note, too, that senders and receivers may differ in their encoding and decoding strategies at the onset of a communicative system [83]. In this, we describe the first step in a process of sign formation: signers must identify specific aspects of the visual signal and ascribe meaning to it. Through use, communicative and articulatory pressures, along with other hallmarks of language change, ultimately constrain what a sign may look like.

## Supporting information

S1 Table Mean lexical parameter values between surveys. (PDF)

S2 Table F-statistics and p-values for between-survey differences in lexical parameter means. (PDF)

S1 File F-tests for feature selection. Available to view/download at https://osf.io/xsp4c/.
(TXT)

S2 File Correlation matrix. Available to view/download at https://osf.io/xsp4c/
(TXT)
