# Supplementary material for: Visual form of ASL verb signs predicts non-signer judgment of transitivity
Source: PLoS One. 2022 Feb 25;17(2):e0262098. doi: 10.1371/journal.pone.0262098 (PMC8880903; doi:10.1371/journal.pone.0262098)
Supplement: S1 Table — (PDF) [file pone.0262098.s001.pdf]

S1 Table. Mean lexical parameter values between surveys

Feature	Survey 1	Survey 2	Survey 3	Survey 4	Survey 5	Survey 6
SignFrequency(M)	4.274	4.545	4.377	4.266	4.462	4.279
Iconicity(M)	3.135	3.224	3.345	3.662	3.834	3.827
SubtLexUSLog10WFa	3.397	3.739	3.266	3.243	3.632	3.367
SignLength(ms)	769.518	669.356	700.7	659.799	635.01	705.005
Minimal Neighborhood Density	737.031	757.788	783.419	774.71	793.75	780.742
Maximal Neighborhood Density	26.781	35.091	33.806	31.355	35.375	34.129
Parameter-Based Neighborhood Density	5.813	5.818	5.613	6.032	7.25	5.129
Sign Type Frequency	290.094	270.636	282.129	270.161	267.813	296.935
Location Frequency	287.188	242.03	265.419	253.29	268.813	291.839
Minor Location Frequency	190.781	129.606	139.903	128.968	164.25	191.355
Selected Fingers Frequency	292.906	317.333	317.452	295.355	361.25	289.097
Flexion Frequency	228.969	315	321.548	371.677	310.688	326.774
Movement Frequency	187.063	216.03	208.677	188.065	220.75	203.935
Handshape Frequency	70.625	94.939	98.258	104.161	107.625	92.065
