# Supplementary material for: Visual form of ASL verb signs predicts non-signer judgment of transitivity
Source: PLoS One. 2022 Feb 25;17(2):e0262098. doi: 10.1371/journal.pone.0262098 (PMC8880903; doi:10.1371/journal.pone.0262098)
Supplement: S2 Table — (PDF) [file pone.0262098.s002.pdf]

S2 Table. F-statistics and p-values for between-survey differences in lexical parameter means

Feature	F	p
SignLength (ms)	0.6886	0.6327
Iconicity (M)	1.6494	0.1490
SubtLexUSLog10WF	1.5895	0.1650
Minimal Neighborhood Density	0.9491	0.4505
Maximal Neighborhood Density	0.5048	0.7724
Parameter-Based Neighborhood Density	0.4441	0.8172
Sign Type Frequency	0.4358	0.8232
Location Frequency	1.3934	0.2286
Minor Location Frequency	0.8324	0.5281
Selected Fingers Frequency	1.4672	0.2025
Flexion Frequency	1.0913	0.3667
Movement Frequency	0.7035	0.6215
Handshape Frequency	1.2499	0.2876
Sign Frequency (M)	0.2577	0.9355
Parameter-Based Neighborhood Density	0.4441	0.8172
